# Micro- and Nano-Systems Developed for Tolcapone in Parkinson’s Disease

**DOI:** 10.3390/pharmaceutics14051080

**Published:** 2022-05-17

**Authors:** Yaquelyn Casanova, Sofía Negro, Karla Slowing, Luis García-García, Ana Fernández-Carballido, Mahdieh Rahmani, Emilia Barcia

**Affiliations:** 1 Department of Pharmaceutics and Food Technology, School of Pharmacy, Universidad Complutense de Madrid, Ciudad Universitaria s/n, 28040 Madrid, Spain; ycasanov@ucm.es (Y.C.); soneal@ucm.es (S.N.); afernand@ucm.es (A.F.-C.); mahdiera@ucm.es (M.R.); 2 Institute of Industrial Pharmacy, Universidad Complutense de Madrid, Ciudad Universitaria s/n, 28040 Madrid, Spain; 3 Department of Pharmacology, Pharmacognosy and Botany, School of Pharmacy, Universidad Complutense de Madrid, Ciudad Universitaria s/n, 28040 Madrid, Spain; karlas@ucm.es; 4 Brain Mapping Lab, Pluridisciplinary Research Institute, Universidad Complutense de Madrid, Ciudad Universitaria s/n, 28040 Madrid, Spain; lgarciag@ucm.es

**Keywords:** tolcapone, microparticles, nanoparticles, PLGA, Parkinson’s disease

## Abstract

To date there is no cure for Parkinson’s disease (PD), a devastating neurodegenerative disorder with levodopa being the cornerstone of its treatment. In early PD, levodopa provides a smooth clinical response, but after long-term therapy many patients develop motor complications. Tolcapone (TC) is an effective adjunct in the treatment of PD but has a short elimination half-life. In our work, two new controlled delivery systems of TC consisting of biodegradable PLGA 502 (poly (D,L-lactide-co-glycolide acid) microparticles (MPs) and nanoparticles (NPs) were developed and characterized. Formulations MP-TC4 and NP-TC3 were selected for animal testing. Formulation MP-TC4, prepared with 120 mg TC and 400 mg PLGA 502, exhibited a mean encapsulation efficiency (EE) of 85.13%, and zero-order in vitro release of TC for 30 days, with around 95% of the drug released at this time. Formulation NP-TC3, prepared with 10 mg of TC and 50 mg of PLGA 502, exhibited mean EE of 56.69%, particle size of 182 nm, and controlled the release of TC for 8 days. Daily i.p. (intraperitoneal) doses of rotenone (RT, 2 mg/kg) were given to Wistar rats to induce neurodegeneration. Once established, animals received TC in saline (3 mg/kg/day) or encapsulated within formulations MP-TC4 (amount of MPs equivalent to 3 mg/kg/day TC every 14 days) and NP-TC3 (amount of NPs equivalent to 3 mg/kg/day TC every 3 days). Brain analyses of Nissl-staining, GFAP (glial fibrillary acidic protein), and TH (tyrosine hydroxylase) immunohistochemistry as well as behavioral testing (catalepsy, akinesia, swim test) showed that the best formulation was NP-TC3, which was able to revert PD-like symptoms of neurodegeneration in the animal model assayed.

## 1. Introduction

Parkinson’s disease (PD) represents the second most prevalent neurodegenerative disorder in the elderly population after Alzheimer’s disease. PD is a disabling progressive disorder presenting tremors, slow movements, stiffness, and postural instability as the neurodegeneration progresses [1]. The incidence of PD is rapidly increasing, it being estimated that it is the fastest growing neurodegenerative disease worldwide [2].

Over the last decades, the main treatment for PD in monotherapy is based on the administration of levodopa, which in the early stages of the disease provides a smooth clinical response. However, after a few years of treatment many PD patients develop motor complications. Regarding complex PD in which patients suffer from moderate to severe disability and cognitive impairment, it can be treated with combination therapy including catechol-O-methyltransferase inhibitors (ICOMTs), along with dopamine (DA) precursors [3].

COMT is a selective and widely distributed enzyme involved in the catabolism of levodopa, with tolcapone (TC) being a selective and potent COMT inhibitor able to slow down levodopa metabolism thereby leading to a prolongation of its effect [4]. TC inhibits COMT activity, in both the brain and the peripheral tissues [5], and has been approved as an adjunctive therapy for PD patients who are treated with levodopa/carbidopa. 

Although several years ago rare reports of severe hepatotoxicity limited its use, a reappraisal of the data for TC in PD has determined that this risk is very small if proper hepatic monitoring is conducted [4]. Moreover, various studies on TC have indicated that the drug may have a wider safety window than previously believed [6,7]. Current recommendations indicate the need to perform tests on liver function before starting the patient on TC and avoiding its use in patients with impaired liver functionality. According to the European Medicines Agency (EMA), EMEA/H/C/000132 [8], in any case, once patients begin treatment with TC, their liver function should be monitored.

TC has a short elimination half-life of around 1.6 to 3.4 h [9,10], with the area under the plasma concentration vs. time curve being dose-dependent [11]. After oral administration, the overall bioavailability of TC is around 65%, the usual dosage schedule being t.i.d. (three times a day). These frequent dosing intervals have an important impact on adherence thereby, resulting in less effective treatments for the disease. It should also be noted that poor treatment adherence/compliance can be a very important problem when managing chronic diseases such as PD. 

For this, the development of controlled release systems can significantly minimize poor patient compliance as dosage intervals can be extended. In this regard, polymeric micro- and nano-systems prepared with biocompatible and biodegradable polymers such as PLGA may enable achieving sustained drug release at the targeted site over long periods of time. 

The potential applications of MPs and NPs for PD therapy have been explored in the last few years for different drugs, such as levodopa [12,13], rasagiline [14,15], puerarin [16], schisantherin A [17], glial cell line-derived neurotrophic factor (GDNF) [18], ropinirole [19], and apomorphine [20]. 

Due to their sizes ranging from 1 to 1000 µm microparticles (MPs), they are unlikely to cross most biological barriers; however, they present the possibility to accurately control the release rate of the encapsulated drugs over long periods of time (hours to months), thereby facilitating less frequent dosing intervals. 

The blood brain barrier (BBB), due to the tight junctions between the endothelial membranes, represents the main obstacle for drugs to enter the CNS. To overcome this fact, polymeric nanoparticles (NPs) are being extensively investigated for their potential use in neurodegenerative diseases as they can facilitate the passage of drugs across the BBB. NPs are sub-micrometer-sized carrier materials designed to improve the biodistribution of encapsulated agents by delivering them more selectively and effectively to the target site [21,22,23].

In our study, two new drug delivery systems are developed for TC, consisting of PLGA microparticles and nanoparticles. After characterization, the best formulations are evaluated in a rotenone-induced animal model of PD.

## 2. Materials and Methods

### 2.1. Materials

Tolcapone (TC) was obtained from Jinan Haohua Industry Co., Ltd., (Jinan Haohua Industry Co., Ltd., Jinan, China); the neurotoxin rotenone (RT) was obtained from Sigma-Aldrich (Madrid, Spain); PVA (polyvinyl alcohol) of Mw = 72 kDa was purchased from Merck (Madrid, Spain); and the polymer PLGA (poly (D,L-lactide-co-glycolide acid) Resomer^®^ RG 502 (ratio 50:50 with inherent viscosity of 0.2 dL/g) was obtained from Evonik Industries (Darmstadt, Germany). The other reagents/solvents used were of analytical grade and obtained from Panreac (Madrid, Spain). Purified water from a Milli-Q filtration system (Merck Millipore, Burlington, MA, USA) was employed in the preparation of solutions/buffers. 

### 2.2. Preparation of TC-Loaded Microparticles

The method used for the preparation of TC microparticles was that of solvent extraction–evaporation from an O/W emulsion. For this, 400 mg of polymer PLGA 502 were weighed and dissolved in 1.5 mL of dichloromethane (DCM) with vortex stirring for 2 min. A fixed amount of TC (70–120 mg) (Table 1) was added to this solution and stirred for 2 min until a homogeneous mixture of the active ingredient was obtained. The dispersion formed was added to 10 mL of 1% PVA and homogenized with a polytron at 5000 rpm for 3 min until the emulsion was formed. Then, the emulsion was transferred to a beaker containing 50 mL of 0.1% PVA solution and kept with gentle agitation for 3 h at room temperature until complete evaporation of the organic solvent. The microspheres were then filtered through 0.45 µm filters and washed with Milli-Q water. Finally, the microparticles were freeze-dried to remove the remaining moisture. All batches of microparticles were prepared in triplicate (Table 1).

### 2.3. Preparation of TC-Loaded PLGA Nanoparticles

Tolcapone nanoparticles were prepared by the nanoprecipitation technique [24], which requires low energy, allowing for the use of an organic solvent such as acetone with better toxicity profile than others [25]. The amount of PLGA 502 used in the preparation of all NPs formulations was 50 mg, and that of TC ranged from 6 mg to 12 mg (Table 1). For this procedure, both the drug (TC) and the polymer (PLGA 502) were dissolved in acetone (4 mL) under agitation (2 min). Then, the solution obtained was added to 0.5% PVA (12 mL) under constant agitation (15 min) to form the NPs. To completely remove the organic solvent, the suspension was evaporated for 2 h at 25 °C and 70 mBar in a Büchi rotavapor-R (BÜCHI Labortechnik AG, Flawil, Switzerland). The resulting suspension was washed with Milli-Q water and centrifuged (Avanti J-301, Beckman Coulter Inc., Brea, CA, USA) at 15,000× *g* rpm to eliminate all PVA. Liophillization of the dispersed solution obtained was performed for 24 h, employing 3% sucrose as a cryoprotectant agent (Lyo Quest^®^, Telsta Technologies S.L, Barcelona, Spain).

### 2.4. Characterization of Microparticles and Nanoparticles

#### 2.4.1. Morphological Characterization and Size Distribution

SEM (scanning electron microscopy) was used to analyze the morphology of the microparticles (MPs) and nanoparticles (NPs) in a JEOL JEM 6335F system (Jeol Ltd., Tokyo, Japan). For this procedure, samples were coated with colloidal gold applied in a cathodic vacuum evaporator. SEM analysis was carried out at 20 KV. Moreover, laser diffraction measurements were carried out in a Microtrac-S3500 analyzer (Micro-trak Inc., Largo, FL, USA) to estimate mean diameters and size distributions of MPs and NPs. Before each measurement, the lyophilized samples were suspended in Milli-Q water and sonicated for 30 s to avoid clumping. Data were expressed as mean diameter and standard deviation (SD).

#### 2.4.2. Calculation of Process Yield and Encapsulation Efficiency

Estimation of encapsulation efficiency (EE%) was performed according to the ratio between the amount of TC encapsulated within the MPs/NPs and the amount of TC used in their preparation according to the following equation:

EE% = amount of TC encapsulated within MPs/NPs(mg) × 100/initial amount of TC used in the preparation of MPs/NPs (mg)

The ratio between the weight of MPs/NPs obtained after preparation and the weight of drug/polymer used was employed for estimating process yield (%).

To determine the amount of TC incorporated within the MPs/NPs, the following procedure was used: 10 mg of MPs/NPs were weighed, to which 1 mL of DCM and 16 mL of ethanol were added to precipitate the polymer. Then, samples were centrifuged at 3000× *g* rpm for 5 min. The supernatant obtained after centrifugation at 5000× *g* for 5 min was then filtered through 0.45 mm filters and analyzed by an HPLC method previously validated by the authors. 

#### 2.4.3. Quantification of Tolcapone by HPLC

Quantification of TC was performed by high-performance liquid chromatography (HPLC). The apparatus consisted of an HPLC Waters chromatograph with a model 510 pump, a model 1490 E UV detector, a 717 auto sampler, and an Empower Login HPLC System Manager Software (Waters Corporation, Milford, MA, USA). A Mediterranean C18 column (5 µm, 250 mm × 4 mm) (Teknokroma S. Coop., Barcelona, Spain) was used. The mobile phase was composed of phosphate buffer:acetonitrile (30:70, *v/v*). The 0.05 M phosphate buffer was prepared from monobasic potassium phosphate (KH_2_PO_4_) and adjusted to pH 2 with phosphoric acid. Before use, the mobile phase was filtered through 0.45 μm filters and degassed. The flow rate was 1 mL/min and the injection volume was 20 μL. For analysis, a wavelength of 268 nm was used with the sensitivity adjusted to 0.250 aufs. All analyses were performed at 25.0 ± 0.5 °C. The method was linear within the concentration range of 0.5–20 µg/mL, with a limit of detection (LOD) of 0.12 µg/mL and limit of quantification (LOQ) of 0.36 µg/mL. 

PLGA did not interfere with the chromatographic method. 

#### 2.4.4. Zeta Potential

A Laser-Doppler anemometry with a Malvern Zetasizer (Malvern Panalytical, Malvern, UK) was employed for measuring the zeta potential of the NPs. Analyses were carried out at 25 °C in aqueous medium with the effective voltage set at 150 V. Briefly, an exact amount (5 mg) of each formulation was placed in a flask, diluted with distilled water (50 mL), and sonicated for a period of 5 min. For zeta potential estimations, each sample was placed in a capillary cell (Cell Enhances Capillary^®^, Malvern Panalytical, Malvern, UK). All formulations were analyzed in triplicate.

#### 2.4.5. In Vitro Release Study

In vitro release of the drug (TC) from all the formulations developed (MPs and NPs) was performed by suspending 20 mg of each formulation in PBS (3 mL). The tests were carried out in a water bath (Memmert, Schwabach, Germany) kept at 37 ± 0.2 °C and constant agitation (100 rpm). At pre-fixed times, all sample volume was withdrawn and the supernatant was removed and filtered through 0.45 μm filters for MPs. For NPs, samples were ultracentrifuged at 15,000 rpm for 20 min and filtered through 0.05 μm filters. The volumes withdrawn were then replaced with fresh medium. Tests lasted 42 days for MPs and 27 days for NPs. Quantification of TC was performed by direct spectrophotometry at 266 nm. In vitro release tests were carried out in triplicate.

### 2.5. Animal Testing

Animal experiments were carried out in male Wistar rats weighing 180–220 g and obtained from Harlan (Harlan France SARL, Gannat, France). Animal experiments complied with the 3R principles (reduction, replacement, and refinement). Animals were housed at 22 ± 2 °C under normal laboratory conditions on a standard light–dark cycle. Food and water were supplied ad libitum. To minimize pain and discomfort, all adequate measures were taken with efforts made to minimize the number of animals used. All experimental procedures were approved by the Ethics Committee for animal testing of the Universidad Complutense de Madrid (permit number PROEX: 14/18) and carried out according to the Spanish RD 1201/2005 regarding the care and use of experimental animals. 

#### 2.5.1. Treatments and Animal Groups

The neurotoxin rotenone (RT) was given intraperitoneally (i.p.) to the animals dissolved in sunflower oil [26]. The following animal groups were included in the study:

- Group 1 (G1): Control group. Animals (n = 8) receiving the vehicles; sunflower oil (subgroup G1A, n = 4) or saline (subgroup G1B, n = 4).
- Group 2 (G2): Animals (n = 8) receiving only the neurotoxin RT (2 mg/kg/day) for 43 days.
- Group 3 (G3): Animals (n = 8) receiving RT (2 mg/kg/day) for 43 days and the amount of MPs equivalent to 3 mg/kg/day of TC every 14 days from day 15.
- Group 4 (G4): Animals (n = 8) receiving RT (2 mg/kg/day) for 43 days and the amount of NPs equivalent to 3 mg/kg/day of TC every 3 days from day 15.
- Group 5 (G5): Animals (n = 8) receiving RT (2 mg/kg/day) for 43 days and TC in saline (3 mg/kg/day) from day 15.

Doses assayed were chosen based upon previous experiments carried out in our laboratory. For this, appropriate amounts of MPs/NPS adapted to animal weight and encapsulation efficiency were injected i.p. MPs/NPs were dispersed in saline for administration. After 44 days, animals were sacrificed by decapitation with a guillotine. 

#### 2.5.2. Body Weight Evaluation

On pre-established times (1, 5, 10, 14, 20, 26, 30, 35, 40, and 44 days) animals were weighed to determine changes occurring throughout the study. 

#### 2.5.3. Behavioral Testing

*Catalepsy test*. This test consists of placing the animal in an unusual posture and then recording the time taken for the animal to correct this posture. In our study, both the “grid test” and the “bar test” were performed to measure catalepsy. Catalepsy tests were performed on days 16, 25, 35, and 44 of the study. After a period of adaptation to the test, animals were placed either on a bar (bar test) situated 10 cm above and parallel from a horizontal base or hung by all four paws (grid test) on a vertical grid (25.5 cm × 44 cm). 

For the bar test, latency with removal of the paw was recorded. For the grid test, the time (latency) needed for the animal to make the first move was recorded. The maximum descent latency was established at 180 s. Catalepsy tests were performed in triplicate.

*Akinesia test*. This test estimates the delay taken for the animal to initiate a movement, also giving information regarding the upper limb motor function. Akinesia test was carried out on days 16, 25, 35, and 44 by estimating the latency in seconds required for the animals to move all four limbs. When latency surpassed 180 s the test was terminated. Before initiating the experiments, animals were adapted to the procedure by placing them for 5 min on an elevated (100 cm) platform (100 × 150 cm). Akinesia tests were carried out in triplicate. 

*Swim-test.* Swimming scores were obtained on days 16, 25, 35, and 44 of the study. The procedure used is that of Haobam et al. [27] with minor modifications. For this, animals were placed in warm water tubs (27 ± 2 °C). The following swim-scores were used: score 0 (hind part sinks with head floating), score 1 (occasional swimming using hind limbs while floating on one side), score 2 (occasional swimming/floating), and score 3 (continuous swimming). Tests were carried out in triplicate.

#### 2.5.4. Histochemical Assessments

*Brain processing*. At the end of the study (44 days), animals were decapitated using a guillotine. Brains were removed, frozen on dry ice, and stored at −80 °C until analysis. Coronal brain sections (30 μm thick) at the level of striatum and substantia nigra were obtained by means of a cryostat (Leica CM1850, Leica Biosystems Nussloch GmbH, Nußloch, Germany). All brain slices were thaw-mounted onto Superfrost Plus slides (Thermo Fisher Scientific, Dreieich, Germany), dried at 36 °C on a hot plate, and then kept frozen at −80 °C.

*Nissl-staining.* For this test, brain samples were fixed with 4% formaldehyde prepared in PBS at pH 7.4. Samples were washed twice with phosphate buffer and submerged in 0.5% cresyl violet acid solution for 30 min. Samples were then washed with distilled water and dehydrated in graded ethanol solutions (70%, 95%, and 100%). Finally, all samples were cleared in xylene (twice, 5 min each) and coverslipped with DPX mounting medium (dibutyl phthalate in xylene) (Sigma-Aldrich, Madrid, Spain). Images of the substantia nigra were obtained by means of a DFC425 digital camera (Leica Biosystems Nussloch GmbH, Nußloch, Germany) coupled to a light microscope (Leitz Laborlux S microscope, Leica Biosystems Nussloch GmbH, Nußloch, Germany).

*TH immunohistochemistry.* A 1:500 dilution of the TH (tyrosine hydroxylase) antibody (Sigma-Aldrich, Madrid, Spain) was added to the samples once fixed, permeabilized with 0.1% Tween 20 in TBS (tris buffered saline), washed, and blocked. After overnight incubation at 4 °C, all slides were washed with TBS/0.1% Tween 20 (3 × 5 min at RT), and then the corresponding secondary FITC (fluorescein isothiocyanate)-labeled antibody (dilution 1:500; Sigma-Aldrich, Madrid, Spain) was added. Thereafter, samples were incubated for 2 h at RT, washed, and mounted with Mowiol aqueous medium. Images were obtained by means of a fluorescence microscope (Leica DM 2000LED, Leica Biosystems Nussloch GmbH, Nußloch, Germany). Digital images were captured using the FITC filter (Leica DFC 3000G, Leica Biosystems Nussloch GmbH, Nußloch, Germany).

*GFAP immunohistochemistry*. Glial fibrillary acidic protein (GFAP) immunohistochemistry was carried out as reported by Garcia-Garcia et al. (Garcia-Garcia et al., 2015). For this, samples were fixed with 4% formaldehyde, washed, permeabilized in TBS/0.1% Tween 20, and blocked in 5% albumin dissolved in TBS. Overnight incubation was carried out at 4 °C with a fluorescent anti-GFAP antibody conjugated with a 1:500 dilution of the Cy3 cyanine dye (Sigma-Aldrich, Madrid, Spain). With this procedure, there is no need for a secondary antibody. The unbound antibody was eliminated by washing the samples. Finally, brain slices were coverslipped with Mowiol mounting medium. Images were observed using a fluorescence digital camera (Leica DFC 3000G, Leica Biosystems Nussloch GmbH, Nußloch, Germany) coupled to a microscope (Leica DM 2000LED, Leica Biosystems Nussloch GmbH, Nußloch, Germany) using the tetramethylrhodamine isothiocyanate (TRITC) filter.

### 2.6. Statistical Analysis

One-way ANOVA was used for analysis, with statistical significance defined as *p* < 0.05 with data obtained from the experiments expressed as mean ± standard error of the mean. All statistical analyses were performed with the Statgraphics^®^ *Plus* v.5.1 software (Statistical Graphics Corporation, Warrenton, VA, USA). Results from the animal behavioral tests were analyzed by means of non-parametric analyses (multifactorial Kruskal–Wallis one-way ANOVA).

## 3. Results and Discussion

Controlled drug delivery systems are very interesting approaches when dealing with active compounds exhibiting short elimination plasma half-lives, non-specific biodistribution, and off-site toxicities as they can facilitate the access of drugs to their specific target sites. When targeting to the CNS, there is a growing interest in the development of micro- and nano-based systems for improving the pharmacological and therapeutic properties of conventional drugs, increasing dosage intervals and reducing adverse peripheral side-effects.

In this work, we have developed two new controlled release systems (microparticles and nanoparticles) for tolcapone (TC) using PLGA 502 as a polymer. PLGA, a copolymer of poly lactic acid (PLA) and poly glycolic acid (PGA), is approved by the FDA [28] and the EMA [8] for its use in pharmaceutical products administered to humans via conventional oral and parenteral routes as well as suspension formulations for implantation without surgical procedures [29], due to its biocompatibility and biodegradability, long clinical experience, favorable degradation characteristics, and possibilities for sustained drug delivery. After administration, PLGA yields to glycolic and lactic acids, which are rapidly cleared from the body after entering the Krebs’ cycle to be degraded into CO_2_ and H_2_O [30,31].

Several TC-loaded PLGA formulations of microparticles (MPs) have been prepared with different amounts of TC ranging from 70 mg to 120 mg (Table 1, formulations MP-TC1 to MP-TC4). Mean values of process yield for all TC-loaded PLGA MPs ranged from 62.33 ± 15.10% to 87.69 ± 7.04%. Encapsulation efficiency (EE) increased with the amount of TC used (70 to 120 mg), with the highest value obtained for the formulation prepared with 120 mg of TC (85.13 ± 2.08%) (Table 1). A successful microparticle system should have a high drug-loading capacity in order to reduce the number of MPs administered, thereby reducing the amount of polymer given.

**Figure 1:**
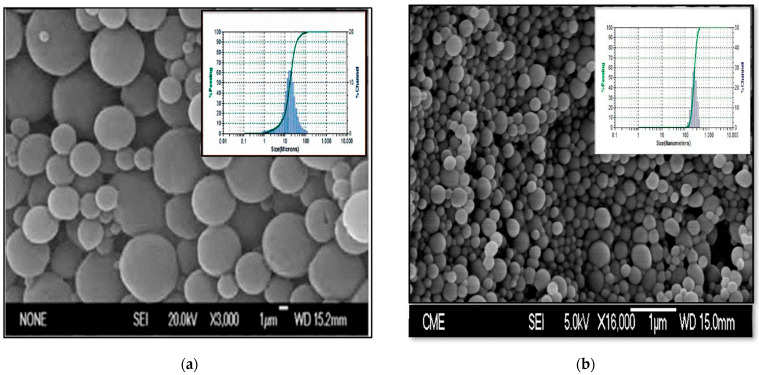
SEM microphotograph of TC-loaded PLGA MPs (formulation MP-TC4) and particle size distribution (**a**). SEM microphotograph of TC-loaded PLGA NPs (formulation NP-TC3) and particle size distribution (**b**). TC (tolcapone).

Mean particle sizes ranged from 16.35 ± 0.10 µm to 27.73 ± 2.59 µm, being in all cases lower than 35 µm (Table 1). From the results obtained, formulation MP-TC4 was selected to perform further studies. This formulation was prepared with 120 mg of TC and presents an EE of 85.13% and mean particle size of 17.00 µm. Figure 1a shows microphotographs of formulation MP-TC4 and its particle size distribution (DLS image).

In our work, we have also developed several TC-loaded PLGA formulations of NPs using different amounts of the drug (Table 1, formulations NP-TC1 to NP-TC4). Taking into consideration the importance of particle size and size distribution regarding drug loading capacity, drug release, and stability of NPs as well as regarding their biological fate within the body, toxicity, and targeting ability, we have determined mean particle sizes of NPs by means of dynamic light scattering (DLS) (Table 1). For all formulations prepared, the mean particle size was around 200 nm, which is suitable for improving the access of TC to the brain. Several studies have demonstrated that NPs prepared with polymers such as PLGA and particle sizes of 250 nm are able to reach the CNS [32,33]. Figure 1b shows microphotographs of formulation NP-TC3 with its particle size distribution. The mean values of process yield for all TC-loaded PLGA NPs ranged from 55.78 ± 16.87% to 75.28 ± 5.73% (Table 1). Mean EE of TC within the NPs ranged from 46.16 ± 5.99% to 56.16 ± 4.65%. Loading efficiency increased as the amount of TC increased from 6 to 10 mg, but not when 12 mg was used. In this case, loading efficiency was similar to that obtained with 10 mg (0.88 mg TC/10 mg of NPs vs. 0.89 mg TC/10 mg of NPs). Higher values of loading efficiency are desirable in order to minimize the number of NPs administered. From the results obtained, formulation NP-TC3 was selected to continue the study. 

**Figure 2:**
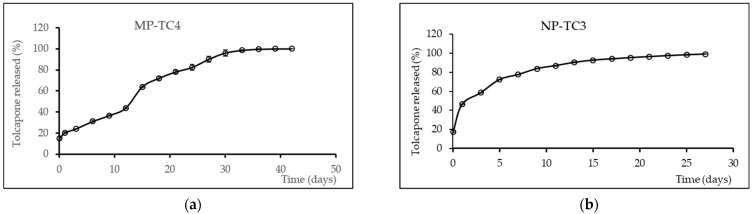
Mean release profiles (±SEM, n = 3) of TC from formulations MP-TC4 (**a**) and NP-TC3 (**b**). TC (tolcapone).

Figure 2a shows the cumulative in vitro release profiles of TC obtained for the microparticle formulation selected, MP-TC4. For PLGA MPs, the release of drugs occurs via diffusion and/or homogeneous bulk erosion of the biopolymer, with the diffusion rate dependent upon drug diffusivity and partition coefficient [34]. Moreover, burst release occurs because of the formation of surface cracks in the MPs, which facilitates their erosion [35]. In our case, the initial burst release of TC from formulation MP-TC4 was low (around 15% within the first hour). High burst release is usually regarded as a negative effect when considering the long-term performance of a drug delivery system. Moreover, this burst release effect leads to undesirable consequences such as more frequent dosing intervals and local toxicity due to the release of high drug concentrations at short times. Burst release has been associated with different chemical, physical, and processing parameters when preparing MPs [36]. For instance, the migration of the drug during the drying process leads to heterogeneous drug distribution, thereby facilitating this burst release. 

For formulation MP-TC4, the burst release was followed by zero-order release kinetics for around 30 days. The mean zero-order release rate constant was 2.13 µg/h. After 30 days, the percentage of TC released was around 95%. This slow release could be explained by the hydrophobic nature of TC, which hinters water diffusion into the MPs, thereby reducing the rate of polymer degradation [37].

Figure 2b shows the in vitro cumulative TC release profiles obtained for the nanoparticle formulation selected (NP-TC3). A burst release of around 18% was obtained within the first hour. At 24 h, 46% of TC was released followed by a slower release for 8 days with mean zero-order release rate constant of 1.77 μg/h/10 mg NPs. Drug release can be affected by particle size. Small particles such as NPs have large surface area-to-volume ratios, being therefore most of the drug associated with small particles that would be at or near the particle surface, thereby leading to faster drug release. 

Zeta potential is a key parameter regarding the surface charge properties of NPs. It reflects the electrical potential of the particles being influenced by their composition and that of the medium in which they are dispersed. It has been demonstrated that NPs with zeta potential values above ±30 mV are stable in suspension as this surface charge prevents the particles from aggregation [38]. Additionally, zeta potential has an influence on the passage of NPs across the BBB. It has been described that, at low concentrations, negatively charged NPS do no influence the BBB passage, but if positively charged interaction with the cell surfaces can occur, thereby facilitating their passage, a stronger immune response can also occur [39]. In our case, the value of zeta potential for the formulation selected (NP-TC3) was −26.32 ± 0.48 mV, being adequate for crossing the BBB. This value can be due to the ionization of the PLGA carboxylic groups, which lead to a negative charge in the surface of the particles. In fact, it has been indicated that NPs prepared with PLGA 502 exhibit a negative zeta potential of around −33 mV [40]. The zeta potential value increased when loading the NPs with TC as a consequence of the modification of the surface due to the incorporation of the drug.

Moreover, previous research studies have demonstrated that nanoparticles prepared with PLGA of sizes around 250 nm were able to cross the BBB [32,33,41]. In our case, NPs presented mean particle sizes of around 200 nm. 

To determine the efficacy of the two new controlled delivery systems developed for TC, the selected formulations (MP-TC4 and NP-TC3) were evaluated in an experimental rotenone-model of PD, which was induced in male Wistar rats. Several studies have demonstrated that, in animal models, RT can induce several PD-like abnormalities including Lewy body formation in the nigral neurons due to systematic inhibition of mitochondrial complex I, oxidative stress, alpha-synuclein phosphorylation and DJ-1 acidification and translocation, proteasomal dysfunction, and nigral iron accumulation [26,42,43].

In our study, behavioral, histological, and immunochemistry tests were carried out in order to determine the efficacy of the formulations developed for TC. 

Regarding mortality in our study, RT was given at a low dose (2 mg/kg/day), which resulted in a mortality rate of 12.3% in group G2 (animals treated only with RT), without any deaths occurring in control animals. This value is similar to that reported by Cannon et al. [44] when using RT (2.75–3 mg/kg/day) and for which 10% mortality occurred shortly after administration of the neurotoxin. In another study, RT was assayed at two dose levels (2 and 2.5 mg/kg), resulting in mortality rates of 6.7% and 46.7%, respectively [30]. 

**Figure 3:**
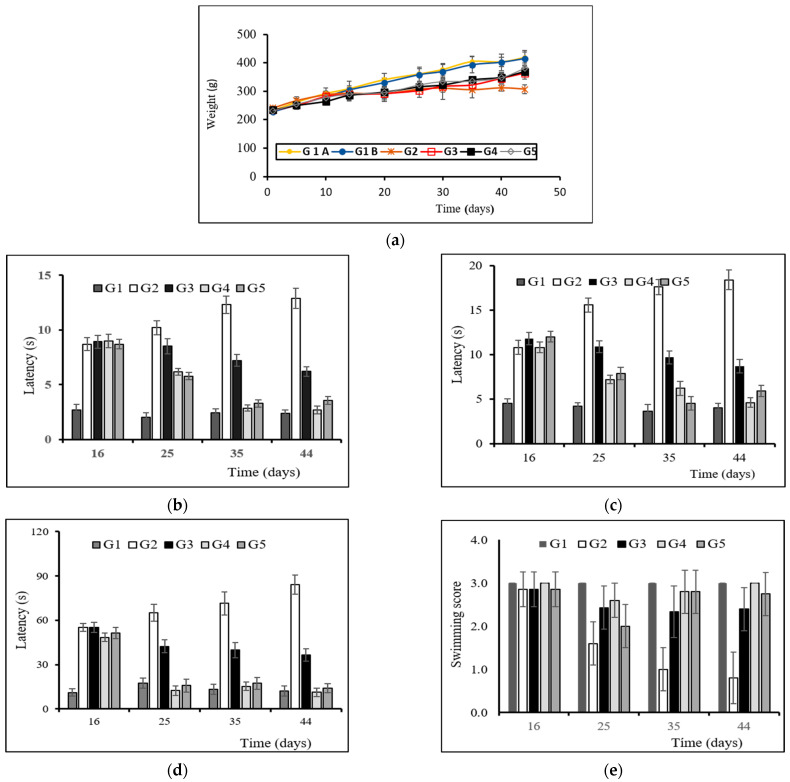
Evolution of rat-body weight throughout the study (44 days) (**a**). Results of the behavioral tests: catalepsy test on bar (**b**) and grid (**c**), akinesia test (**d**), and swim-test (**e**). Animal groups: control subgroups (G1A and G1B); RT-treated control group (G2); RT-treated animals also receiving formulation MP-TC4 (G3), formulation NP-TC3 (G4), and TC in saline (G5). RT (rotenone), TC (tolcapone).

Figure 3a shows the evolution of body weight with time. Control groups (G1A and G1B) corresponding to animals receiving the vehicles (sunflower oil or saline) experienced a gradual and steady weight gain throughout the study, with non-significant differences (*p* > 0.05) observed between both subgroups. Animals treated with RT showed a slight weight increase for the first 10 days, with no gain in weight thereafter. Animals treated with TC-containing formulations showed a steady weight gain throughout the study.

The mean results obtained in the catalepsy test (bar and grid) are depicted in Figure 3b,c, with non-statistically significant differences found between both control subgroups (G1A and G1B), indicating that the vehicles used did not have any influence on the test. The neurotoxin RT induced an increase in latency, both in the bar and grid test (group G2) as compared to control animals that was reverted by the active compound (groups G3, G4, and G5). Among these groups, the best results correspond to animals receiving the NP formulation (group G4), for which non-statistically significant differences were found when compared to group G1 at the end of the study period (*p* > 0.05). 

Akinesia results demonstrated non-statistically significant differences within control subgroups G1A and G1B (Figure 3d). Latency was clearly prolonged with RT (group G2), resulting in a latency value of around 7 times higher in group G2 with respect to G1 at the end of the study period (44 days).

Reversion of akinesia was observed in groups G3, G4, and G5 at times 25, 35, and 44 days. After 44 days, the latency values obtained for groups G4 and G5 were similar to that of the control group, without any statistically significant differences (*p* > 0.05) found between both groups (G4 and G5). In the case of group G5, this could be attributed to the fact that TC was given once daily in solution, indicating that the formulation exhibited a very rapid effect on the brain. Regarding formulation G4, which corresponds TC-loaded PLGA NPs, the results obtained confirm the ability of NPs to deliver TC to the brain and maintain TC levels in the CNS between dosing intervals. 

Swim-tests were performed to evaluate the animals’ overall motor capacity/deficit. The results obtained are depicted in Figure 3e. At all times, assayed animals corresponding to the control group (G1) obtained the highest values (score = 3). The swimming ability of the animals treated with RT (group G2) markedly decreased when compared to the control group (*p* < 0.05). At the end of the study, all animal groups receiving TC (G3, G4 and G5) showed high swimming scores (score > 2), but only group G4 resulted in swim scores equal to control animals. 

**Figure 4:**
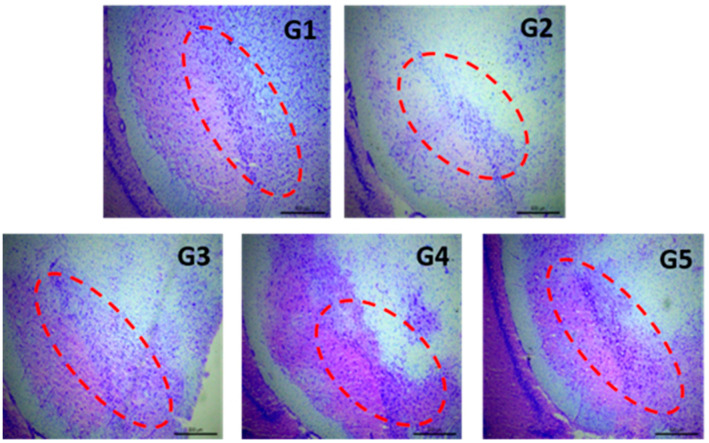
Representative Nissl-staining (cresyl violet) of nigral neurons from brain sections (substantia nigra, 30 µm) corresponding to all animal groups. Control group (G1); RT-treated control group (G2); RT-treated animals also receiving formulation MP-TC4 (G3), formulation NP-TC3 (G4), and TC in saline (G5). RT (rotenone), TC (tolcapone).

The histological method of Nissl staining is widely used to study the morphology and pathology of neurons, as the stain used binds to DNA from nuclei and RNA from cytoplasm of cells, allowing study of the cytoarchitectony of the brain [45]. To determine the effects of the neurotoxin RT and the different formulations on neuronal loss in the SNpc, Nissl staining was performed, with the images obtained shown in Figure 4. Rotenone is a highly lipophilic compound that readily crosses the BBB, thereby allowing for the development of PD-like symptoms in animals. After systemic administration, RT induces progressive degeneration of the nigrostriatal pathway, resembling that occurring in PD patients [44,46]. The results obtained in our study confirm that i.p. injection of RT reproduced the characteristic behavioral and histopathological features of PD, including loss of neuronal cells in the SNpc [47,48,49]. The administration of TC in solution (group G5), or encapsulated within PLGA MPs (group G3) or PLGA NPs (group G4), prevented the cell death induced by RT in SNpc. This effect was more marked when TC was given encapsulated within PLGA NPs, indicating an improved access to the brain. 

The glial fibrillary acidic protein (GFAP) is one of the fibrous proteins forming the intermediate filaments of the intracellular cytoskeleton. This glial response is a source of trophic factors and can protect against the formation of reactive oxygen species (ROS) and glutamate [50], taking into consideration that PD is associated with a glial response mainly composed of activated microglial cells and, to a lesser extent, reactive astrocytes. Moreover, when a brain insult occurs, a slow astrocytic response maintains microglia activation, eventually leading to a chronic brain lesion [51]. 

**Figure 5:**
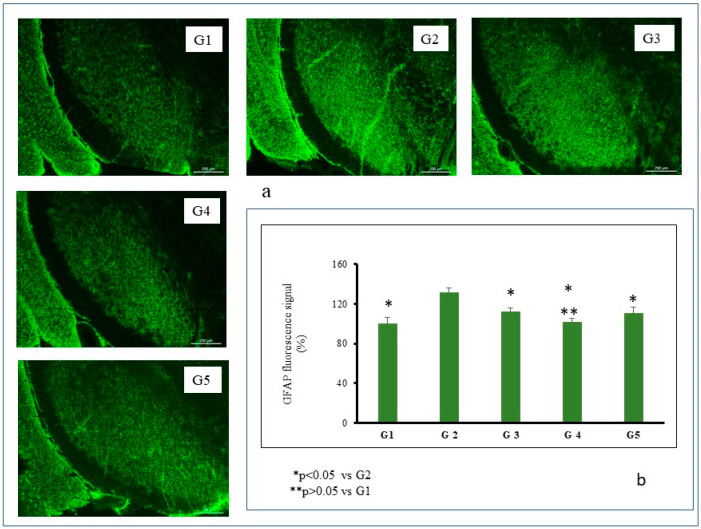
Representative GFAP fluorescence (**a**) and GFAP fluorescence signal (**b**) in coronal brain slices at the level of the substantia nigra for all animal groups. Control group (G1); RT-treated control group (G2); RT-treated animals also receiving: formulation MP-TC4 (G3), formulation NP-TC3 (G4), and TC in saline (G5). GFAP (glial fibrillary acidic protein), RT (rotenone), TC (tolcapone).

For this reason, and in order to determine whether there is a possible astrocytic activation associated with the neurodegeneration caused by RT, non-quantitative immunohistochemical studies of GFAP were performed (Figure 5). The administration of RT at a dose of 2 mg/kg/day for 43 days (group G2) produced intense gliosis. These results are in agreement with those obtained by other authors that described an intense astrocytic activation caused by RT, both in vitro [49] and in vivo [47]. This astrocytic response includes hypertrophy and thickened cell bodies, as occurred in group G2, features which are typical of reactive astrogliosis [52]. Activation of astrocytes causes brain damage due to an increase in the production of ROS [53] and the release of pro-inflammatory cytokines [54,55]. 

As seen in Figure 5b, intense gliosis occurred in the SNpc after 43 days of RT administration (group G2). This astrocytic response was almost reverted in all animal groups receiving TC either encapsulated (groups G3 and G4) or in solution (group G5). However, the response was completely reverted only when TC-loaded PLGA NPs were given to the animals. For this group, non-statistically significant differences were found when compared with control animals (*p >* 0.05). 

A significant alteration occurring in PD is the degeneration of dopaminergic neurons, which leads to a reduction of striatal dopamine levels. Dopamine is synthesized in dopaminergic neurons of the SNpc area, stored in synaptic vesicles and released in the striatum to exert its physiological function [41]. Taking into consideration that tyrosine hydroxylase (TH) catalyzes the formation of levodopa, which is a rate-limiting step for the biosynthesis of dopamine, PD can be considered as a TH-deficiency syndrome of the striatum. For this, the TH immunohistochemical test is useful as it allows quantifying the degree of loss of dopaminergic cells in postmortem brains of individuals diagnosed with PD. Moreover, the use of RT as a neurotoxin has been associated with an observable reduction of TH-immunoreactive neurons in the SNpc [56,57,58]. 

**Figure 6:**
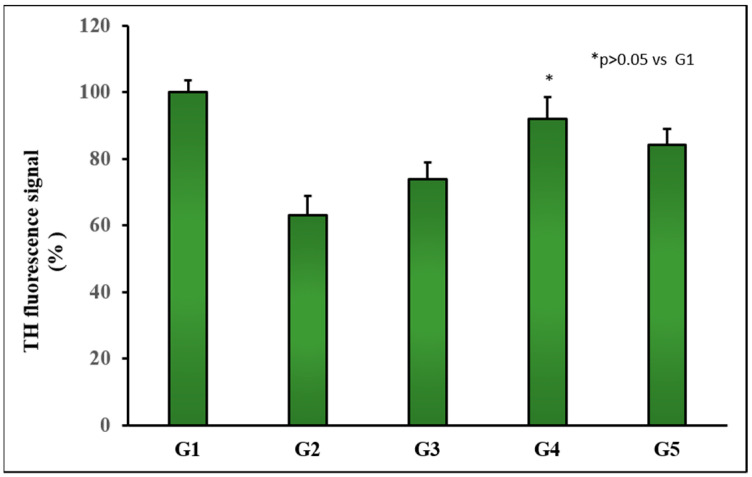
Representative TH fluorescence signal in coronal brain slices at the level of the substantia nigra for all animal groups. Control group (G1); RT-treated control group (G2); RT-treated animals also receiving formulation MP-TC4 (G3), formulation NP-TC3 (G4), and TC in saline (G5). TH (tyrosine hydroxylase), RT (rotenone), TC (tolcapone).

Figure 6 depicts the results of fluorescence intensity obtained in our study for the different animal groups. It can be seen that TH immunoreactivity in SNpc was decreased in group G2, which corresponds to animals treated with daily doses of RT. This reduction was partially reversed in animals receiving TC-containing formulations, with the strongest reversion obtained with TC-loaded PLGA NPs (group G4). For this formulation, non-statistically significant differences were found when compared to the control group (*p >* 0.05), thereby demonstrating the potential interest of this new drug delivery system developed for TC.

## 4. Conclusions

In vivo evaluation of both controlled delivery systems developed for TC consisting of biodegradable PLGA 502 microparticles (formulation MP-TC4) and nanoparticles (formulation NP-TC3) resulted in satisfactory results, with the nanoparticulate system being able to almost completely reverse the rotenone-induced neurodegeneration in the animal model assayed. With both formulations, extending the dosage intervals could be achieved.

## Figures and Tables

**Table 1:** Formulations prepared. MP (microparticles). NP (nanoparticles). TC (tolcapone). EE (encapsulation efficiency).

Formulation	Amount of TC (mg)	Process Yield (%) ± SEM	EE (%) ± SEM	Mean Particle Size ± SEM
MP-TC1	70	62.33 ± 15.10	73.92 ± 10.17	27.73 ± 2.59 μm
MP-TC2	80	78.30 ± 11.96	79.63 ± 3.55	23.05 ± 3.53 μm
MP-TC3	100	84.58 ± 5.75	83.17 ± 4.82	16.35 ± 0.10 μm
MP-TC4	120	87.69 ± 7.04	85.13 ± 2.08	17.00 ± 0.04 μm
NP-TC1	6	55.78 ± 16.87	56.16 ± 4.65	197.39 ± 43.19 nm
NP-TC2	8	75.28 ± 5.73	55.99 ± 21.41	202.08 ± 48.70 nm
NP-TC3	10	70.35 ± 14.19	53.69 ± 9.09	182.59 ± 23.94 nm
NP-TC4	12	73.29 ± 4.50	46.16 ± 5.99	210.20 ± 7.92 nm

## Data Availability

Not applicable.
